# The posttraumatic stress interview for children (KID-PIN): development and validation of a semi-structured interview of PTSD symptoms among displaced children in the Middle East

**DOI:** 10.7717/peerj.12403

**Published:** 2021-12-15

**Authors:** Hawkar Ibrahim, Claudia Catani, Frank Neuner

**Affiliations:** 1 Department of Psychology, Clinical Psychology and Psychotherapy, Bielefeld University, Bielefeld, North Rhine-Westphalia, Germany; 2 vivo International, Konstanz, Germany

**Keywords:** PTSD, Children, Assessment, Arab spring, Kurd, Arab, Iraqi, Syrian

## Abstract

**Background:**

In populations affected by mass disaster such as armed conflict and displacement, children are at risk of developing mental ill-health, in particular post-traumatic stress disorder (PTSD). Valid and reliable screening instruments are needed to assess the severity of PTSD symptoms among children and to identify individuals in need of treatment.

**Method:**

In the context of an ongoing war in the Middle East, we developed the KID-PIN as a semi-structured interview for PTSD symptoms that can be administered by trained paraprofessionals. To achieve a culturally and contextually appropriate instrument, the development was based on open-ended interviews with affected children and involved both local and international experts. Using the KID-PIN and instruments for constructs associated with PTSD, 332 Iraqi and Syrian displaced children were interviewed. A subset of the sample (*n* = 86) participated in validation interviews based on experts applying the Clinician-Administered PTSD Scale for DSM-5—Child/Adolescent Version (CAPS-CA-5).

**Results:**

The KID-PIN demonstrated excellent internal consistency (Cronbach’s alpha = 0.94) with good convergent validity. Confirmatory factor analyses of the KID-PIN showed an acceptable fit with the DSM-5 and other common models; the best fit was reached with the Hybrid model. Receiver operating characteristic analyses indicated that the cut-off score of 28 or higher on the KID-PIN is the optimum cut-off for a probable PTSD diagnosis.

**Conclusion:**

The utility of the newly developed KID-PIN as a screening instrument for PTSD in children is supported by the measure’s high internal consistency and good convergent and structural validity, as well as its diagnostic accuracy.

## Introduction

According to the most recent report by United Nations High Commissioner for Refugees (UNHCR), the global population of displaced people due to war, conflict, and persecution has substantially increased in the last decade. UNHCR’s statistics documented that, by the end of 2018, 70.8 million people were forced to flee their homes worldwide, and more than half of them were under the age of 18 (United Nations High Commissioner for Refugees, 2019). The current conflicts in Iraq and Syria have significantly contributed to this increase. After the events of the Arab Spring, Syria and Iraq have faced several multi-sided armed conflicts including civil and international wars, between current governments, opposition armies, extremist groups, and neighboring countries. As a result of these conflicts, more than half of Syria’s population and millions of Iraqis fled war to find safety (United Nations High Commissioner for Refugees, 2018).

A large number of studies on children’s mental health showed that displaced children are at high risk for developing mental health conditions (Rousseau, 1995; Fazel & Stein, 2002; Bronstein & Montgomery, 2011). Post-traumatic stress disorder (PTSD) is one of the most common psychological disorders among war-affected and displaced children (Fazel, Wheeler & Danesh, 2005; Attanayake et al., 2009; Tam, Houlihan & Melendez-Torres, 2017). Although there is no systematic survey on trauma and PTSD among Iraqi internally displaced children after the current crisis, available research evidence suggests that Syrian children are exposed to a wide range of war-related events with high rates of PTSD symptoms. Gormez et al. (2018), who investigated the prevalence of traumatic events and psychopathology among Syrian school children in Turkey, documented that 70% of them have witnessed explosions or gun battles and more than half of the children had lost at least one close person during the war. As a consequence, 69% of the children were suffering from anxiety-related disorders and 18.3% were diagnosed with PTSD. Perkins et al. (2018) examined mental health problems among Syrian students in Syria and found that more than half of their sample had at least one probable psychological disorder, with PTSD the most common (35.1%), followed by depression (32.0%), and anxiety (29.5%). Similar prevalence rates were found by other studies among Syrian children in a reception camp in Germany and in Jordan (Soykoek et al., 2017; Yonis et al., 2019).

The surveys mentioned above are a clear account of the mental health needs of children in war-affected regions such as Iraq and Syria. At the same time, these studies illustrate the challenges of conducting clinical research in such contexts. However, due to some methodological limitations in trauma and PTSD studies on Iraqi and Syrian children, their results should be interpreted with caution. First, since almost all current mental health instruments (Pynoos et al., 2015; Sachser et al., 2017; Foa et al., 2018; Kaplow et al., 2019) have been developed in high-income countries, most often in the United States (US), it is common practice to apply *ad-hoc* translations of PTSD scales for the assessment of symptoms across the world. Specifically, despite the current crisis in Syria and Iraq, no study has applied a PTSD instrument that had been systematically adapted and validated in a Middle East population. In addition, given that the transference of a cut-off score from one context, language, and population to another can lead to substantial over- or underestimation of prevalence rates (Ibrahim et al., 2018b), the prevalence rates of PTSD in these studies may not be accurate. Second, the instruments applied in the studies mentioned above do not reflect the current diagnostic criteria for PTSD in the fifth edition of the Diagnostic and Statistical Manual of Mental Disorders (DSM-5) (American Psychiatric Association, 2013), which is the most widely used standard of current mental health criteria in research. Third, beyond the instruments’ validity, almost all of these studies focused on children currently enrolled in school. However, many children in conflict regions have dropped out of school due to war and displacement. International organizations have observed low rates of school enrolment among Syrian children. A recent report by Save the Children (2017) concluded that one out of three school-aged children in Syria is no longer in school and others are at a high risk of dropping out of school. Moreover, despite several existing valid and reliable instruments for evaluating traumatic events and PTSD among children and adolescents, the Child PTSD Symptom Scale for DSM-5 (Foa et al., 2018), and the UCLA Child/Adolescent PTSD Reaction Index for DSM-5 (Kaplow et al., 2019) reflect DSM-5 symptoms and criteria for PTSD. Still, it is not certain that these instruments can be used in other cultural and working settings as evidence regarding their cross-cultural validity is sparse, and psychometric information about these instruments is generally limited to non-refugees and western samples.

The aim of the current study reported in this paper was to address the existence of a validation gap in the lack of contextually suitable instrument for assessment of PTSD symptoms among children affected by the current conflict in Iraq and Syria. Thus, this study sought to contribute to establishing a valid and reliable screening tool for refugee children. There is an urgent need for such evaluations according to a recent systematic review on trauma and mental health screening tools for refugee children and youth (Gadeberg & Norredam, 2016; Gadeberg et al., 2017).

To meet the requirements of the contextually valid PTSD instrument, rather than translating and adapting an instrument that is currently available, we decided to develop a new instrument in Kurdish and Arabic languages that would more accurately meet the requirements of the context. For this purpose, bilingual Kurdish and Arabic mental health experts developed the items of this instrument based on both an understanding of the meaning of the DSM-5 criteria of PTSD as well as open-ended discussions on these symptoms with affected children. With this procedure we aimed to construct formulations that were comprehensible and accessible for children with diverse backgrounds in terms of culture and education while maintaining the international comparability and transference of concepts and findings.

To account for the variance of literacy in this population and to ensure the correct understanding of the items by the respondents, the Posttraumatic Stress Interview for Children (KID-PIN) was developed as a structured interview to be administered by trained paraprofessionals. To establish validity, we compared the factor structure of the instrument with DSM-5 and other alternative models that have been suggested for underlying dimensions of PTSD symptoms, namely, the dysphoria (Simms, Watson & Doebbelling, 2002), dysphoric arousal (Elhai et al., 2011), anhedonia (Liu et al., 2014), externalizing behaviors (Tsai et al., 2015), and Hybrid model (Armour et al., 2015). More recently, based on exploratory factor analysis among a large sample of displaced Iraqi and Syrian adults, Ibrahim et al. (2019) proposed a new model *“anhedonia and affect model*” and found its superiority on the DSM-5 model (see Table 1 for the item mappings for PTSD factor structure cross different models). Moreover, we assessed the associations between KID-PIN with related constructs and compared the findings with expert ratings of PTSD using an instrument (CAPS-CA-5) commonly considered a gold-standard instrument in high-income countries.

**Table 1:** Item mapping for PTSD factor structure models assessed in Iraq and Syrian children.

KID-PIN items	DSM-5	Dysphoria	Dysphoric arousal	Externalizing behaviors	Anhedonia	Hybrid	Anhedonia and affect
1. Intrusive thoughts	R	R	R	R	R	R	R
2. Nightmares	R	R	R	R	R	R	R
3. Flashbacks	R	R	R	R	R	R	R
4. Emotional cue reactivity	R	R	R	R	R	R	R
5. Physical cue reactivity	R	R	R	R	R	R	R
6. Avoidance of thoughts	A	A	A	A	A	A	A
7. Avoidance of reminders	A	A	A	A	A	A	A
8. Trauma-related amnesia	NACM	D	NACM	NACM	NACM	NA	NCBA
9. Negative beliefs	NACM	D	NACM	NACM	NACM	NA	NCBA
10. Distorted blame	NACM	D	NACM	NACM	NACM	NA	NCBA
11. Persistent negative emotional state	NACM	D	NACM	NACM	NACM	NA	NCBA
12. Lack of interest	NACM	D	NACM	NACM	An	An	An
13. Feeling detached	NACM	D	NACM	NACM	An	An	An
14. Inability to experience positive emotions	NACM	D	NACM	NACM	An	An	An
15. Irritable/ angry	AR	D	DA	EB	DA	EB	NCBA
16. Recklessness	AR	AR	DA	EB	DA	EB	NCBA
17. Hypervigilance	AR	AR	AA	AA	AA	AA	NCBA
18. Exaggerated state	AR	AR	AA	AA	AA	AA	NCBA
19. Difficulty concentrating	AR	D	DA	DA	DA	DA	NCBA
20. Sleep disturbance	AR	D	DA	DA	DA	DA	NCBA

**Note:**

R, re-experiencing; A, avoidance; NACM, negative alterations in cognitions and mood; AR, alterations in arousal and reactivity; D, dysphoria; DA, dysphoric arousal; AA, anxious arousal; EB, externalizing behaviors; An, anhedonia; NA, negative affect; NACM, negative alterations in cognitions and mood.

The rationale of the KID-PIN and this study builds upon the assumption that PTSD is a universal psychological disease that results from similar psychological and biological processes that occur after traumatic events. However, we expect that the verbal and behavioral expression of symptoms will be shaped by the cultural context and align with common ways to verbalize and express stress and discomfort in a specific language. Since the factorial structure is closer to the psychopathological processes than the single items, we expect to find a symptom structure similar to those found in other contexts. At the same time, we do not consider a translation of an instrument that was developed and validated in a different culture, such as the CAPS-CA-5, as the gold standard that can be used to establish criterion validity. However, we perceive that some research and clinical practice questions, such as epidemiological or clinical research depending on case definitions, demand international comparability. For this reason, we checked the KID-PIN values against the most commonly used international standard, the CAPS-CA, by determining the best fitting cut-off value and the resulting agreement in the case definition.

The current study aimed to develop a psychometrically sound instrument (KID-PIN) for measuring PTSD among traumatized children, which could be applicable in low resource and post-conflict settings such as Iraq and Syria. The study attempts to establish psychometric adequacy for KID-PIN by testing the internal consistency, establishing convergent and structural validity by correlation and factor analysis, and establishing diagnostic utility by Receiver operating characteristic (ROC) analysis.

## Methods

### Development of the KID-PIN

#### General outline of the KID-PIN

The goal of the KID-PIN was to develop an assessment instrument of PTSD symptoms related to specific traumatic events based on DSM-5 diagnosis criteria for PTSD. To assess multiple traumatic events and allow the determination of the most impactful traumatic event, the KID-PIN follows a checklist of traumatic event types that should be targeted to the specific population. Considering the lived context of Syrian and Iraqi refugees, we utilized the War and Adversity Exposure Checklist (WAEC), which had been specifically developed to assess traumatic experiences both in the context of war events as well as outside a war context (Ibrahim et al., 2018a).

#### Response format

We developed a simple response format that would allow those administering the instrument to determine the severity of symptoms by accounting for a minimum frequency of symptoms. For each symptom, the KID-PIN asks for the frequency of symptoms during the past month on a simple three-point scale (0 = never; 1 = once; and 2 = more than 1 time). For symptoms that were reported as 2 (more than 1 time) the severity of the symptom is rated on a five-point scale ranging from 0 (not at all) to 4 (extremely). The severity of symptoms that had been reported only occurring one time or less during the past month was rated as 0. Due to limited literacy skills as well as the young age of the children, and to get as accurate a response as possible, the severity rating was supported by visual cues representing different severities, besides the reading and verbal explanation of the rating scale anchors. Children were asked to indicate the level of a liquid in a glass as a description of how much they were bothered by a given symptom in last month (empty glass: corresponding to not at all; less than half-empty corresponding to a little bit; half-empty glass: corresponding to moderately; more than half-empty corresponding to quite a bit; and a glass full of liquid corresponding to being extremely bothered by the symptom).

#### Development of symptom descriptions

##### First: open ended interview

After obtaining informed consent from their parents as well as their own assent, 17 displaced Kurdish and Arab children (47.1% and 52.9%, respectively) between 8 and 16 years (*M* = 10.94, *SD* = 2.72), were interviewed by six locally trained clinical psychologists and social workers using a semi-structured interview. The interviews were conducted at the participants’ homes in a separate room without the presence of their parents and other family members and took between 45 to 65 min.

The interview started with basic demographic questions such as age, gender, education, ethnic and religious affiliation, and four open-ended questions about personal migration histories as well as lifetime traumatic events with a particular focus on potential traumatic events that could take place during war and displacement (see Table S1). Participants were then asked to report the psychosocial and physical impact of these traumatic events on their wellbeing. Participants reported a variety of mental symptoms (such as depressed mood 52.9%, fear 41.2%, bad concentration 35.3%, upsetting dreams 29.4%, and anger 11.8%) and physical symptoms (such as weakness 41.2%, headache 29.4%, stomachache 23.5%, and bed wetting 5.9%). In order to gain more specific information regarding symptoms related to PTSD, the symptom clusters of PTSD were explained to participants, and they were asked if they had ever had any similar symptoms (see Table S1 for the details of the open-ended questions). Thereafter, based on the participants’ wordings, 20 items that reflected DSM-5 criteria for PTSD were generated and had to be rated on a five-point scale (ranging from “never = 0” to “extremely = 4”).

##### Second: expert panel

In order to evaluate the face-validity and child-friendliness of the wording of the scale, eight local professionals active in the areas of mental health and child development were invited to an expert panel. The participants on the expert panel were between 30 and 51 years (*M* = 38.12, *SD* = 7.66) old and had more than 2 years of experience in the field of psychotraumatology (*M* = 5, *SD* = 2.72). The majority (62.5%) of experts were psychotherapists, and the others were psychologists and psychiatrists (25% and 12.5%, respectively). Participants had either a Master of Science (MSc.) or a doctorate in psychology, and they had either an academic position (25%), clinical position (12.5%), or both (62.5%), and all of them were experienced in developing culturally appropriate psychological instruments for the Kurdish and Arabic context. Panel participants were asked to provide their opinion about the extent to which the drafted scale adequately captured the meaning of the symptoms of PTSD. Also, they were asked to share their own opinion about the wording and semantics of each item. All experts agreed that the drafted scale had excellent face validity. Based on their professional viewpoints some items were altered to be more age appropriate. For example, experts suggested adding the word “*Kabus*-nightmare” as a further explanation for disturbing dreams in item 2, since, in this cultural context, some children use this term as an expression for disturbing or scary dreams. For item 19 “problems with concentration” experts suggested adding some examples that could be used with both children who had been enrolled in school and those who had not, such as “being distracted in class” for children in school and “being distracted when someone tells you a story” for children who were not in school at the time of administration.

##### Third: pilot study

A pilot study was undertaken in order to assess the feasibility of the scale, as well as to evaluate possible language barriers, level of comprehension, and the scale’s internal consistency. In addition to the KID-PIN scale, several other instruments were used including a basic demographic questionnaire, the WAEC (Ibrahim et al., 2018a) for assessing the number of experienced lifetime traumatic events, and the Short Mood and Feelings Questionnaire (SMFQ)—Child Version (Angold et al., 1995) for examining depression symptoms. The pilot study was conducted among 21 participants (47.6% female and 52.4% male), aged between 8 and 16 years (*M* = 11.52, *SD* = 2.33). Results showed that the PTSD scale had good internal consistency (total items Cronbach’s α = 0.92, intrusion Cronbach’s α = 0.91, avoidance, Cronbach’s α = 0.92, negative alterations in cognition and mood, Cronbach’s α = 0.79, trauma-related alterations in arousal and reactivity α = 0.74). Moreover, results showed that the sum score of the scale was positively correlated with the number of experienced traumatic events (*r*_*s*_ = 0.61, *p* < 0.001) and with the SMFQ sum score (*r*_*s*_ = 0.75, *p* < 0.001). Regarding the administration of the PTSD scale, locally trained interviewers reported that they had spent between 20 and 30 min for each child (*M* = 22.21, *SD* = 3.42). In addition, the local interviewers also confirmed that the wording and semantics of items were easy for participants to understand.

### Psychometric evaluation

#### Participants

Two rounds of interviews were conducted to evaluate the KID-PIN’s psychometric properties, including screening interviews and validation interviews. Firstly, using KID-PIN screening interviews were conducted with 332 Iraqi and Syrian children and adolescents between 8 and 16 years of age (*M* = 12.67, *SD* = 2.08). Then, about half of them (48.2%) were chosen to participate in the validation (diagnostic) interviews. Although 145 of them agreed to participate (response rate 90.6%), only 86 of them could be successfully involved in the validation interviews due to constraints around the limited time period of this study since scheduling was difficult during the schooling period. The participants in the validation subsample were carefully balanced in terms of gender, ethnicity, nationality, and language of the interview (sociodemographic information of both samples presented in Table 2).

**Table 2:** Sociodemographic information and traumatic experiences.

			Full study sample	Validation sample
Interview language N (%)				
		Kurdish	182 (54.8)	47 (54.7)
		Arabic	150 (45.2)	39 (45.3)
Sex, *N* (%)				
		Male	160 (48.2)	45 (52.3)
		Female	172 (51.8)	41 (47.7)
Religion, *N* (%)				
		Muslim	310 (93.4)	81 (94.2)
		Yazidi	22 (6.6)	5 (5.8)
Ethnicity, *N* (%)				
		Kurd	182 (54.8)	47 (54.7)
		Arab	150 (45.2)	39 (45.3)
Nationality, *N* (%)				
		Iraqi	174 (52.4)	44 (51.2)
		Syrian	158 (47.6)	42 (48.8)
Age, mean (SD)^a^^,^^b^			12.67 (2.08)	12.93 (2.07)
Formal education, mean (SD)^a^^,^^c^			4.96 (2.16)	5.09 (1.91)
Number of siblings, mean (SD)			5.45 (3.07)^d^	5.67 (2.87)^e^
Number of lifetime displacements, mean (SD)			1.03 (0.19)^f^	1.02 (0.15)^g^

**Notes:**

a In year.

b Score range: 8–16.

c Score range: 0–10.

d Score range: 0–27.

e Score range: 1–16.

f Score range: 1–3.

g Score range: 1–2.

#### Procedure

##### Sampling and interviewers

The data of the psychometric evaluation was drawn from a multi-informant survey of parents and their children that was carried out between March and April 2019. Fifteen trained Bachelor level psychologists and social workers who were fluent in Kurdish and Arabic languages conducted screening interviews with 332 Iraqi and Syrian children and youths and both of their parents in Arbat camp. Arbat camp is located in the Sulaymaniyah Governorate in the Kurdistan Region of Iraq (KRI) and hosts displaced people from different national (Iraqi and Syrian), ethnic (Kurd and Arab) and religious backgrounds (Muslim and Yazidi). In order to avoid conflicts between ethnics and religious groups, the camp administration divided displaced people in this camp into three subcamps, and each subcamp was subdivided into six or seven sections. Following the example of our previous work in the same camp (Ibrahim et al., 2018a, 2018b, 2019), a pragmatic sampling approach was carried out. A random selection of households was chosen using a spin-the-pen method to determine a direction and a computer-generated sequence of random numbers to select houses in the directions. Out of all children that fulfilled the inclusion criteria within one household, one child was randomly chosen to participate.

Children were eligible for participation if they were at least 4 years old at the time of war and displacement (the probable age for remembering early childhood events; Eacott, 1999) and they were between 8–16 years old at the time of the interview. Participation depended on parental consent. Since the survey aimed to obtain triad data (father-mother-child), both parents needed to agree to their own and their child’s participation. Local interviewers visited households, sat with both parents and one randomly chosen child per family, explained the research study and its aims. After obtaining individual verbal informed consent, face-to-face interviews were conducted separately with both parents and their children in private spaces in or near participants’ homes.

Due to sociopolitical and historical reasons, people in the northern regions of Syria and Iraq speak a variety of languages and it is not possible to determine a common primary language for any given group of people. Thus, participants were free to choose between the Kurdish languages Kurmanji and Sorani or Arabic for their interviews. Although Kurdish Kurmanji has generally been the mother tongue for the Kurdish participants in this sample, they were raised and educated in different languages and dialects. Before their displacement, the Syrian Ba’ath party prohibited the Kurdish language from being taught in the education system. As a result, Syrian youths were educated in Arabic, and after their displacement, the majority of Syrian Kurdish sought refuge in the Sulaymaniyah and Erbil governorates in the KRI, where Kurdish Sorani dialect is an official language. All Arab participants decided to be interviewed in Arabic, and 90.51% of Kurds were interviewed in Kurdish Kurmanji, the remaining participants were interviewed in Sorani.

In the second stage, eight expert clinical psychologists (four Iraqi psychologists with fluency in Arabic and Kurdish and four German psychologists) carried out validation interviews. Local experts had at least a MSc. in clinical psychology with a minimum of 4 years in clinical research and/or clinical experience with severely traumatized populations, including displaced people, fire, and genocide survivors as well as victims of human trafficking, family, and gender-based violence. All local experts were university lecturers at the department of clinical psychology at Koya University in the KRI, and they had more than 4 years’ experience in teaching trauma-related subjects. The international experts also held at least a MSc in clinical psychology, and they were from the department of clinical psychology and psychotherapy at Bielefeld University, Germany. The international experts were trained in clinical diagnostics with survivors of war form diverse backgrounds. Local experts were fluent in the language of the study’s participants, and the international experts conducted the validation interviews through trained interpreters. All expert interviewers were trained in the application of the CAPS-CA-5. During the survey, participants were informed about the validation interviews. Within 2 to 20 days after participation in the survey, trained social mobilizers visited the participants’ home and provided information about the nature and scope of the validation interviews. Then, after receiving the parents’ and children’s consent to participate, social mobilizers scheduled validation interviews with the subsample of 86 participants. Validation interviews were conducted in a private space, usually in participants’ homes. In order to avoid any potential bias, members of the validation team (social mobilizers, expert interviewers, as well as their interpreters) were blind to the results of the screening interviews.

##### Protection and safety

Research into victimization is necessarily delicate, and all respondents were considered to be potentially vulnerable. Therefore, we took some essential steps for the protection of the respondents and the staff. In collaboration with local NGOs, the camp administration, the Directorate of Social Affairs (DoSA) in the Ministry of Labour and Social Affairs, and the protection office in the Joint Crisis Coordination Centre (JCC) in the Ministry of Interior, a protection and referral system was established for those participants who were in need of psychological support. Second, the study was conducted upon the principle of informed consent, meaning that participants were informed about their right to withdraw from the research and to refuse any questions if they did not wish to answer without penalty. They also received debriefing information about the study and its aims as well as data management and confidentiality. However, due to the skepticism and mistrust of the population towards authorities and the potential legal consequences of signed forms (Ibrahim & Hassan, 2017), we relied on obtaining verbal rather than written informed consent through reading standardized written consent information sheets to the respondents. Verbal consent was documented by the interviewers. Third, the quality of the assessments’ administration was ensured by using locally trained interviewers who held at least a B.Sc. in clinical psychology or social work and 2 years of prior experience with traumatized people. Fourth, to examine the quality of work, ethical concerns, the safety of participants, as well as emotional support and enhancing self-care for interviewers, a weekly individual supervision with each interviewer was conducted by the first author. During the supervision sessions, supervisor and supervisee addressed the challenges and barriers of data collection and the potential impacts of secondary exposure to trauma. Furthermore, open group discussions with interviewers were conducted once every 2 weeks during data assessment. Ethical approval for the study was obtained from the ethics committee of Bielefeld University in Germany (reference number: EUB 2015-046) and the ethics committee of Koya University in the KRI (reference number: SHETC-1). Moreover, local government departments, including the JCC and DoSA, also approved the study and its procedure.

#### Measures

##### Potentially traumatic events

Adverse and traumatic life events were evaluated by applying the WAEC (Ibrahim et al., 2018a). The WAEC is a self-report checklist that consists of 26 items assessing the number of adverse experiences related to family and organized violence, and general lifetime traumatic experiences such as sexual violence, accidents, or natural disasters. The internal consistency of WAEC among the full sample as well as the validation subsample was good (Cronbach’s α = 0.83, and 0.85, respectively).

##### Depression symptoms

The child version of SMFQ (Angold et al., 1995) was utilized for examining depression symptoms. SMFQ is a non-diagnostic, self-report instrument that consists of 13 items rated on a three-point Likert scale (0, never; 1, sometimes; 2, always). The sum-score of SMFQ was calculated by summing up all items with a higher sum-score indicating higher levels of depressive symptoms. In the current analysis, the SMFQ had a good internal consistency (Cronbach’s α = 0.88).

##### PTSD diagnosis

In the validation interviews, PTSD symptoms and diagnosis were assessed with the semi-structured interview CAPS-CA-5 (Pynoos et al., 2015), which is considered a gold standard instrument for measuring DSM-5 PTSD symptoms. The CAPS-CA-5 examines the severity of total PTSD symptoms and its sub-clusters (reexperiencing, avoidance, negative alterations in cognition and mood, and hyperarousal) based on symptoms’ frequency and intensity on a five-point Likert scale ranging from 0 “absent” to 4 “extreme/incapacitating”. Symptoms count as present if they are rated as 2 “moderate/threshold” or higher. A PTSD diagnosis was obtained following the DSM-5 standards, which require at least one endorsed symptom of re-experiencing and avoidance, at least two symptoms of negative alterations in cognition and mood, hyperarousal, and the presence of these symptoms for at least 1 month with clinically significant distress or functional impairment.

In the current study, the CAPS-CA-5 had a high level of internal consistency (Cronbach’s α = 0.93) with an excellent convergent validity through testing the relationship between sum scores of CAPS-CA-5 and WAEC (*r*_s_ = 0.67, *p* < 0.001).

#### Data management

All data were de-identified and entered into the database using SPSS (version 25; IBM, Armonk, NY, USA) and cross-checked. In order to avoid missing data, multiple checking steps were taken. Firstly, interviewers were instructed to check their forms immediately after each interview before leaving participants’ homes. Second, every single questionnaire was checked by another interviewer for addressing missing data. Third, during fieldwork, two experienced clinical psychologists carefully reviewed each questionnaire for missing data. If any missing data was found in the second and third steps, the interviewers re-visited the participant’s home to ask for the missing information. The rates of missing data in the second and third step were 2% and 0.5%, respectively.

#### Data analysis

The normality assumption was tested using Kolmogorov–Smirnov and Shapiro–Wilk tests as well as visual inspection of the histogram, stem-and-leaf plot, boxplot, and quantile–quantile plot (Ghasemi & Zahediasl, 2012). Results showed that the sum score and most of the items of the KID-PIN and CAPS-CA-5 in the pilot, screening, and validation studies were not normally distributed. Therefore, Spearman correlation was applied to test convergent validity and Mann–Whitney U tests were conducted in order to compare differences between groups.

The internal consistency of each scale was evaluated by calculating Cronbach’s alpha. The diagnostic accuracy of KID-PIN compared to CAPS-CA-5 was assessed with Receiver Operating Characteristic (ROC). Receiver Operating Characteristic is a graphical display that visualizes the performance diagnostic tests by plotting sensitivity (true positives) on the y-axis against specificity (true negatives) on the x-axis. Moreover, ROC provides the overall accuracy of the diagnostic test through Area Under the Curve (AUC) (Akobeng, 2007; Pintea & Moldovan, 2009). The optimal cut-off score for diagnosing PTSD was determined by choosing a well-balanced value between sensitivity, specificity, positive, and negative predictive values.

In order to assess the structural validity of KID-PIN, confirmatory factor analyses (CFA) were conducted. Due to the violation of the assumption of normality in the data, a maximum likelihood estimation method with Satorra–Bentler’s (S_B) adjustments was used (Satorra & Bentler, 1988, 1994). The overall model fit was examined using multiple fit indices including Tucker–Lewis Index (TLI) (Tucker & Lewis, 1973), Comparative Fit Index (CFI) (Bentler, 1990), and Root Mean Square Error Of Approximation (RMSEA) (Steiger & Lind, 1980; Browne & Cudeck, 1993). The index criteria for an acceptable model fit were: CFI/TLI > 0.90, RMSEA [90% CI] ≤ 0.06 (lower value <0.05, and upper value <0.08), SRMR < 0.08 (Bentler, 1992; Hu & Bentler, 1999; Cheung & Rensvold, 2002; Schermelleh-Engel, Moosbrugger & Müller, 2003; Kline, 2011). Since Chi-square difference tests cannot be applied in non-nested model comparisons, the models were compared using Akaike Information Criterion (AIC) (Akaike, 1974), and Bayesian information criterion (BIC) (Schwarz, 1978). A model with lower AIC and BIC values indicates a better model fit (van de Schoot, Lugtig & Hox, 2012). According to Raftery (1995), ΔBIC values < 10 is significant support for a better-fitting model. ROC and CFA analyses were conducted using Stata 16 (StataCorp, 2019) and IBM SPSS Statistics for Macintosh, Version 25.0 (IBM, Armonk, NY, USA) was used for the other analyses.

## Results

### Traumatic events

Participants in the full sample reported between 0 and 20 traumatic event types (*M* = 4.45, *SD* = 3.83); 91.9% of them had reported at least one traumatic event. Being deprived of food and/or water due to war or flight (20.2%), seeing a dead body (apart from funerals) or a rotting corpse (12.3%), and witnessing a murder (12%), were the most frequently reported traumatic events. Iraqi children and youth were exposed to significantly more traumatic events than Syrian children (*M* = 17.01 *vs*. 9.30, *P* < 0.001). Children who participated in the validation study reported between 1 and 20 traumatic events (*M* = 5.41, *SD* = 3.85) in the screening interviews, while in the validation interviews, they reported between 0 and 21 traumatic events (*M* = 4.84, *SD* = 4.40).

### Descriptive statistics for CAPS-CA-5

The mean score of CAPS-CA-5 total severity was *M* = 8.94 (*SD* = 12.39). Following the DSM-5 scoring rules, 36% of participants meet the B criteria for PTSD, the C, E, and D criteria were met by 27.9%, 18.6%, and 14% of the respondents, respectively; 10.5% met full diagnostic criteria for PTSD.

### Descriptive statistics and internal consistency of KID-PIN

The means, standard deviation, and item-total correlation for each item are presented in Table 3 The Cronbach’s alpha value for KID-PIN full scale and its sub-scales ranged between good and excellent (see Table 4)

**Table 3:** Item-level descriptive statistics for KID-PIN.

Item	M	SD	Item-total correlation	Skewness	Kurtosis	
1. B1	1.78	1.65	0.68	0.15	−1.66	
2. B2	1.23	1.57	0.60	0.84	−0.90	
3. B3	1.15	1.47	0.60	0.90	−0.70	
4. B4	1.91	1.77	0.73	0.05	−1.80	
5. B5	1.50	1.70	0.66	0.50	−1.50	
6. C1	1.91	1.73	0.75	0.04	−1.76	
7. C2	1.76	1.71	0.68	0.20	−1.71	
8. D1	1.38	1.68	0.51	0.60	−1.38	
9. D2	0.43	1.10	0.50	2.48	4.89	
10. D3	0.43	1.20	0.43	2.59	4.99	
11. D4	1.33	1.66	0.70	0.72	−1.22	
12. D5	1.02	1.55	0.52	1.09	−0.54	
13. D6	1.08	1.58	0.68	0.99	−0.72	
14. D7	1.13	1.62	0.65	0.95	−0.84	
15. E1	1.14	1.50	0.51	0.90	−0.78	
16. E2	0.08	0.49	0.25	6.94	51.53	
17. E3	0.65	1.30	0.55	1.77	1.56	
18. E4	1.38	1.64	0.67	0.59	−1.39	
19. E5	0.73	1.29	0.45	1.61	1.19	
20. E6	1.01	1.52	0.70	1.20	−0.20	

**Table 4:** Internal consistency and scale-level descriptive statistics for KID-PIN.

Scales	Number of items	M	SD	Score range	Observed range	Cronbach’s α
KID-PIN	20	23.03	19.67	0–80	0–72	0.92
Criterion B	5	7.57	6.36	0–20	0–20	0.83
Criterion C	2	3.66	3.27	0–8	0–8	0.89
Criterion D	7	6.80	7.15	0–28	0–28	0.80
Criterion E	6	5	5.32	0–24	0–21	0.73

### Convergent validity

In the validation sub-sample, a positive correlation was found between the KID-PIN sum score, CAPS-CA-5 total severity, and the number of experienced traumatic events (*r*_*s*_ = 0.40, *r*_*s*_ = 0.54, respectively, *p*_s_ < 0.001). Results from Mann–Whitney U test showed that participants who met PTSD diagnostic criteria based on CAPS-CA-5 had higher scores on the KID-PIN (children without PTSD: *M* = 20.96, *SD* = 18.54, children with PTSD: *M* = 40.77, *SD* = 21.21, Mann–Whitney U = 161, *P* < 0.001 two-tailed).

Regarding the convergent validity of the KID-PIN among the screening sample, a significant positive correlation was found between KID-PIN and depression symptoms, as well as the number of traumatic types (*r*_*s*_ = 0.61, *r*_s_ = 0.63, respectively, *p*_s_ < 0.001), suggesting good convergent validity.

### Structural validity

To examine the structural validity of KID-PIN, the factor structure of the KID-PIN was compared with the DSM-5 model as well as with six different alternative models for PTSD symptom’s structure (Table 1). Results showed that all models including the DSM-5 model demonstrated an acceptable fit of the data (CFIs and TLIs > 0.90, RMSEAs < 0.06, SRMRs < 0.06). The Hybrid model was identified as the best-represented model for the current study. In the non-nested model comparison, the Hybrid model was slightly better than the Anhedonia model (ΔBIC = 7.835), and it was significantly better than other models (ΔBICs < 10). Moreover, all goodness-of-fit indexes (Table 5) and standardized estimates for factor loadings (Table 6) were higher for the Hybrid model.

**Table 5:** Fit indices for DSM-5 and six alternative models.

Models	Goodness of fit indexes						
	S-B χ^2^ (df)	CFI	TLI	RMSEA	SRMR	AIC	BIC
DSM-5	305.32 (164)***	0.925	0.913	0.051	0.056	17,435.467	17,686.606
Anhedonia and affect	303.64 (164)***	0.926	0.914	0.051	0.056	17,426.870	17,678.009
Dysphoria	310 (164)***	0.922	0.910	0.052	0.056	17,444.586	17,695.725
Dysphoria Arousal	302.54 (160)***	0.924	0.910	0.052	0.055	17,432.960	17,699.319
Externalizing behaviours	283.94 (155)***	0.931	0.916	0.050	0.053	17,399.374	17,684.759
Anhedonia	280.17 (155)***	0.933	0.918	0.049	0.053	17,389.257	17,674.642
Hybrid	261.22 (149)***	0.940	0.924	0.048	0.051	17,358.585	17,666.801

**Note:**

*** *p* < 0.001.

**Table 6:** Standardized estimates for factor loadings.

Items	DSM-5	Anhedonia and affect	Dysphoria	Dysphoria arousal	Externalizing behaviours	Anhedonia	Hybrid
1. B1	0.869	0.859	0.859	0.860	0.860	0.860	0.861
2. B2	0.796	0.795	0.796	0.795	0.794	0.793	0.793
3. B3	0.776	0.776	0.776	0.773	0.774	0.773	0.773
4. B4	0.882	0.882	0.882	0.882	0.883	0.884	0.885
5. B5	0.755	0.755	0.755	0.756	0.754	0.755	0.754
6. C1	0.959	0.956	0.959	0.957	0.957	0.953	0.953
7. C2	0.943	0.946	0.943	0.945	0.945	0.949	0.949
8. D1	0.566	0.560	0.561	0.568	0.565	0.579	0.572
9. D2	0.646	0.669	0.653	0.652	0.651	0.690	0.687
10. D3	0.563	0.590	0.576	0.574	0.570	0.599	0.595
11. D4	0.799	0.804	0.799	0.810	0.815	0.835	0.843
12. D5	0.648	0.692	0.651	0.648	0.648	0.693	0.694
13. D6	0.723	0.800	0.730	0.731	0.734	0.790	0.794
14. D7	0.637	0.698	0.643	0.639	0.632	0.707	0.703
15. E1	0.608	0.607	0.608	0.638	0.853	0.668	0.873
16. E2	0.336	0.333	0.336	0.354	0.462	0.368	0.452
17. E3	0.648	0.633	0.655	0.641	0.650	0.650	0.648
18. E4	0.766	0.761	0.772	0.776	0.779	0.780	0.782
19. E5	0.571	0.565	0.563	0.571	0.519	0.567	0.518
20. E6	0.737	0.772	0.718	0.735	0.681	0.759	0.682

### Diagnostic utility

**Figure 1:**
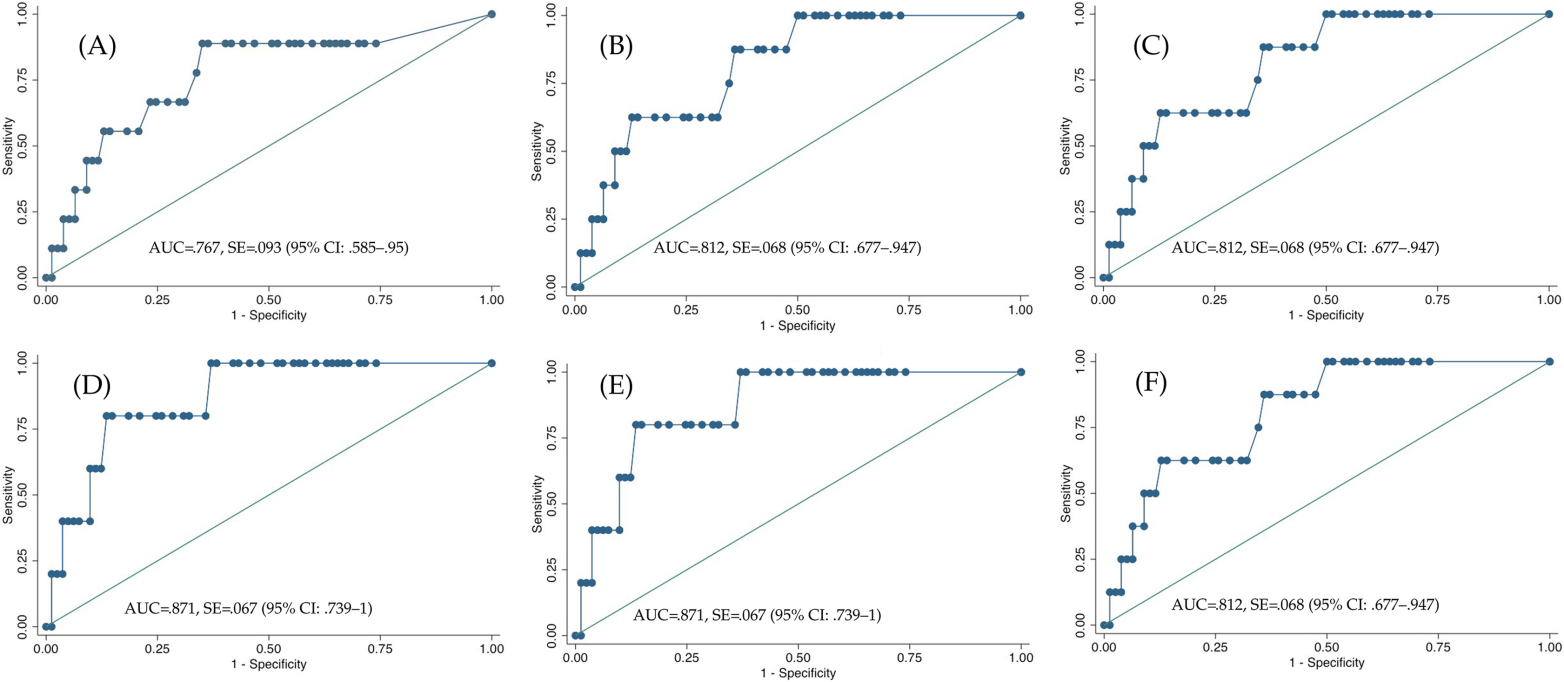
Receiver operating characteristic curves of KID-PIN using different cutoff scores. (A) PTSD criteria only. (B) Cut-off score ≥ 27 (C) Cut-off score ≥ 28 (D) Cut-off score ≥ 29 (E) Cut-off score ≥ 28.5 (F) Cut-off score ≥ 27.5.

The ROC analysis showed that the KID-PIN had a good diagnostic accuracy compared to CAPS-CA-5 (AUC = 0.767, SE = 0.093 (95% CI [0.585–0.950])). Using ROC for determining an optimal cut-off score for probable PTSD diagnosis, we found a cut-off score greater than or equal to 28 as the value with the highest balance between sensitivity, specificity, positive and negative predictive values (AUC = 0.812, SE = 0.068 (95% CI [0.677–0.947])); see Fig. 1, Table 7).

**Table 7:** Performance of the KID-PIN against the CAPS-CA-5 in different cutoff scores.

Statistic	Cut-off score ≥ 27	Cut-off score ≥ 27.5	Cut-off score ≥ 28	Cut-off score ≥ 28.5	Cut-off score ≥ 29	
Sensitivity (%)	88.89	87.5	88.89	80	77.78	
Specificity (%)	63.64	64.1	64.94	64.2	66.24	
Positive predictive value (%)	87.5	20.0	87.5	12.1	80.0	
Negative predictive value (%)	62.8	98.0	64.1	98.1	64.2	
Diagnostic efficiency (%)	65.11	64.04	66.27	62.92	65.11	
Prevalence (%)	9.3	8.98	9.3	5.61	5.8	

## Discussion

The current study aimed to develop and test an instrument for the assessment of PTSD symptoms among children and youth based on DSM-5 diagnosis criteria for PTSD. To meet the needs of the mental health care system in a low- to middle income country such as Iraq, with its limited availability of highly educated psychologists and psychiatrists on the one side and a large proportion of children with limited literacy skills on the other side, we developed this instrument for use by trained BSc-level psychologists and social workers. The results from comprehensive evaluations including experts’ face validity, pilot study, screening, and validation studies suggest that KID-PIN is a reliable and valid scale for measuring PTSD symptoms among children and youths who experienced traumatic events (see Table S2, a translated English version of KID-PIN).

Rather than translating an existing instrument that had been developed in the US, we applied a careful procedure in the development of the KID-PIN that involved expert knowledge on mental health, human development, and local cultural expressions of symptoms. Given the absence of a gold standard for the assessment of PTSD in this context, we tested the instrument against a range of criteria to estimate the reliability and validity of the assessment.

The KID-PIN proved to be a reliable instrument, since it demonstrated excellent internal consistency for the total scale and good internal consistency for its subscales. Consistent with previous studies (Hafstad et al., 2014; Sachser et al., 2017), we found that the self-destructive behavior item had the lowest item-total correlation and was the least frequently reported symptom. Out of 86 children and youths, only two participants (2.4%) had scored two or higher on this item. Previous studies found that endorsing reckless or self-destructive behavior symptom was related to being male (Carmassi et al., 2014) and the severity of PTSD symptoms (Contractor et al., 2017). Consistent with these findings from the literature, our results point in a similar direction. We found that both of these participants were male, and they reported higher severity of PTSD symptoms. Still, due to the small number of participants reporting this symptom, we cannot draw a definite conclusion about the association between gender, the severity of PTSD symptoms, and endorsing reckless behavior symptoms.

In terms of convergent validity, we found a moderate correlation of *r*_s_ = 0.40 between the KID-PIN total score and the severity rating based on the CAPS-5-CA. While this finding indicates that the two instruments assess related constructs, this finding implies that the variables are not identical. Although the CAPS-CA-5 is commonly considered to be a gold-standard instrument for the measurement of PTSD in an international perspective, we have to emphasize that, so far, the CAPS-CA-5 had not been validated either in English or in other languages. Therefore, it is unclear if this moderate correlation is attributed to a limited validity of the KID-PIN or to the validity of CAPS-CA-5. In addition, the relatively long time period of 2 to 20 days (*M* = 7.74, *SD* = 5.54) occurred between the administration of both instruments must also be considered. While such a delay mirrors the practice of a screening procedure that would be later followed by a clinical assessment, it is not an optimal timespan for the assessment of convergent validity. Both symptom change as well as test–retest reliability were factors that probably decreased correlation between the KID-PIN and CAPS-CA-5. In addition to the KID-PIN’s agreement with the CAPS-CA-5, we found a correlation of the expected magnitude (*r*_*s*_ = 0.54) with the number of traumatic event types, which indicates good convergent validity.

We found evidence that the construct measured by the KID-PIN is structurally equivalent to a universal PTSD concept measured in other cultures and settings. In the absence of a gold-standard comparator for the assessment of PTSD, we consider the structural validity of the instrument to be an important indicator of the equivalence of the KID-PIN with other international PTSD instruments. The results of a series of confirmatory factor analyses showed that the DSM-5 model provided an acceptable fit to the data. However, the convergence with an alternative factor structure, the so-called Hybrid was even higher. The hybrid model was proposed by Armour et al. (2015) and consists of seven factors: intrusions, avoidance, negative affect, anhedonia, externalizing behaviours, anxious arousal, and dysphoric arousal. This model was developed based on the combination of features of both six-factor models: anhedonia (Liu et al., 2014) and externalizing behaviours (Tsai et al., 2015). For a comprehensive review of the hybrid model, see Armour et al. (2015). Our finding regarding the superiority of the hybrid model over other models is consistent with an increasing number of studies on the dimensions of DSM-5 PTSD with adults and children (Wang et al., 2015; Sachser et al., 2018). Similar to our analysis, in almost all these studies, the hybrid model showed the best fit with the data and was superior to the DSM-5 model, which, in turn, confirms that the concept measured with the KID-PIN is structurally related to the concepts measured with international PTSD instruments.

Even though there is not sufficient evidence to accept the CAPS-CA-5 as the gold-standard globally, we assume that, for the international comparability of prevalence rates, an agreement with a diagnosis established with the CAPS-CA-5 is desirable. We specified a cut-off score for the KID-PIN with the aim of maximizing the agreement with the CAPS-CA-5 diagnosis using ROC analyses. The KID-PIN performed well (AUC = 0.767) in the diagnostic discrimination between PTSD cases and PTSD non-cases. The cut-off of 28 was found to provide the highest accuracy cut-off for probable PTSD diagnosis. With this cut-off score, we reached the highest level of sensitivity (88.89%) and moderate specificity (64.94%).

## Strengths and limitations

With the methods chosen for the development of the KID-PIN and the subsequent psychometric evaluation we aimed to achieve a high external validity. For the development and validation, we drew on an unselected, non-clinical sample of children with diverse cultural, ethnic, and religious backgrounds to represent war-affected children in the Middle East. A comprehensive approach was applied to develop a practical and psychometrically sound instrument that can be administrated by trained psychologists, social workers, and other aid workers for screening PTSD symptoms and identifying those who need further clinical assessments. Some limitations of the present study should be mentioned. First, the restricted sample size of the validation study limited the statistical power of the analysis. Most notably, the limited sample size did not allow us to analyze the validity of each single language version separately. Second, in the absence of an adapted, valid, and reliable version of CAPS-CA-5 in Kurdish and Arabic languages, we relied on the English version of CAPS-CA-5 as a proxy of an international standard. Third, the convergent validity of KID-PIN was examined with few variables (*e.g*., trauma experience, PTSD based on CAPS-CA-5, and depression). Fourth, the validation study was restricted to the measurement of PTSD and depression, and no discriminant validity could be determined.

## Conclusion

The current study captures the development of a practical and psychometrically sound instrument for screening PTSD symptoms among children and youths. Findings showed that the KID-PIN had high internal consistency and diagnostic accuracy, with good convergent and structural validity. Based on the present analyses, the KID-PIN is a valid and reliable instrument to be used as a screening tool for PTSD with children (aged 8–16 years) by clinicians as well as trained paraprofessionals.

## Supplemental Information

10.7717/peerj.12403/supp-1 Supplemental Information 1 Raw data.

10.7717/peerj.12403/supp-2 Supplemental Information 2 List of questions asked during the open-ended interview.

10.7717/peerj.12403/supp-3 Supplemental Information 3 The English translated version of KID-PIN.

10.7717/peerj.12403/supp-4 Supplemental Information 4 Questionnaire.

## List of abbreviations

AUC: Area Under the Curve
CAPS-CA-5: Clinician-Administered PTSD Scale for DSM-5 - Child/Adolescent Version
KID-PIN: Posttraumatic Stress Interview for Children
PTSD: Post Traumatic Stress Disorder
ROC: Receiver operating characteristic
SMFQ: Short Mood and Feelings Questionnaire – Child Version
UNHCR: United Nations High Commissioner for Refugees
WAEC: War and Adversity Exposure Checklist

## Additional Information and Declarations

### Data Availability

The following information was supplied regarding data availability: The raw data are available in the Supplemental Files.
