# Supplementary material for: The posttraumatic stress interview for children (KID-PIN): development and validation of a semi-structured interview of PTSD symptoms among displaced children in the Middle East
Source: PeerJ. 2021 Dec 15;9:e12403. doi: 10.7717/peerj.12403 (PMC8684325; doi:10.7717/peerj.12403)
Supplement: Supplemental Information 2 [file peerj-09-12403-s002.docx]

# Supplementary Tables

| Supplementary Table 1  List of questions asked during the open-ended interview |
| --- |
| 1. Gender: ___________ 2. Age: ___________ year old. 3. Formal education: ___________(years) 4. Nationality: ___________ 5. Ethnicity: ___________ 6. Religious affiliation: _________ 7. Where did you live before coming here? ___________ 8. Can you tell me how you came here? 9. We have learned that people who have fled their homes had experienced a lot of stressful events, can you tell me what you have experienced during times of war and migration? 10. Which of these events that you mentioned earlier had more impact on you? 11. Can you tell me how this specific event impacted you? (do you have any physical or mental symptoms related to this event? can you tell me how you felt after this event?) 12. Sometimes people who experienced stressful events in their life, such as bad events during war or times of displacement, have some psychological symptoms. I am going to tell you more about these symptoms and how they look. I would like to know if you have ever had any such kind of symptoms and how they are manifesting themselves with you.     1. First of all, sometimes the memories about the bad events suddenly come back to the mind of people who experienced, witnessed, or learned about the particular stressful event, sometimes people feel like they are back to the moment that stressful events took place, and sometimes they have bad dreams or nightmares. Moreover, sometimes people feel certain physical symptoms when someone or something reminds them of a stressful event. Now, I would like to know if you have ever had similar kinds of symptoms about this practical traumatic event that you described previously as a most impactful event in your life. And if you have any of these symptoms, can you tell me how you describe them?     2. Second, sometimes people who experienced, witnessed, or learned about a particular stressful event want to avoid anything like people, places, or activities who remind them about the bad event. Also, sometimes people try to withhold expressing their emotions about the bad thing that happened to them, or they don’t want to think or speak about it. Now, I would like to know if you have ever had these kinds of symptoms about this practical traumatic event that you described previously as a most impactful event in your life. And if you have any of these symptoms, can you tell me how you describe them?     3. Third: sometimes, people who went through negative life events cannot easily remember some important parts of this event, do you remember all the details of this specific event that you mentioned earlier? Or do you think that there are some features of this event that you have forgotten? Moreover, sometimes people have a bad mood after experiencing such adverse events, and they don't enjoy doing things that they enjoyed previously, they feel isolated, and they may blame themselves or others for causing this bad event. Have you ever had any similar feelings and thoughts? If yes, can you tell me how?     4. Fourth: After experiencing adverse life events, sometimes some people get angry very easily and quickly, and sometimes even small things make them angry. Moreover, after traumatic events some other people are purposely doing things that could cause them harm. Did you ever observe any aggression in yourself after experiencing that particular event that you described? Also, sometimes after a traumatic event, some people may become super watchful. For example, even if they are in a safe environment, they are always scanning their environment for possible dangers or threats. Have you ever experienced this kind of state? If yes, can you tell me how? |
