# Supplementary material for: The posttraumatic stress interview for children (KID-PIN): development and validation of a semi-structured interview of PTSD symptoms among displaced children in the Middle East
Source: PeerJ. 2021 Dec 15;9:e12403. doi: 10.7717/peerj.12403 (PMC8684325; doi:10.7717/peerj.12403)
Supplement: Supplemental Information 4 [file peerj-09-12403-s004.docx]

| The English translated version of KID-PIN | | | | | | | | | | |
| --- | --- | --- | --- | --- | --- | --- | --- | --- | --- | --- |
| Sometimes when people face distressful and dangerous events, some problems and symptoms appear. Now I will ask you about these problems and symptoms to find out the extent to which you have such issues. I hope you answer me frankly, and I would like to remind you that there are no right or wrong answers; instead, these questions are about your emotions, feelings, and behavior in the last month.  **Note**: To answer these questions, tell the participant: “From all the stressful events you have experienced, witnessed or heard, keep in your mind the most stressful one that had the most impact on you, for example, an event that you are not able to forget, then answer my questions while thinking about that single event | | | | | | | | | | |
| # | Questions | Symptom Occurrence | | | Symptom Intensity | | | | |  |
|  |  | Never | Once | More than  one time | Not  at all | A little  bit | Moderately | Quite  a bit | Extremely |  |
| 1 | In the past month, have you remembered images, sounds, or memories related to the stressful event that you described as the most stressful one, whether you want to or not? | 0 | 1 | 2 | 0 | 1 | 2 | 3 | 4 |  |
| 2 | In the past month, have you seen any bad or disturbing dreams (nightmares)? | 0 | 1 | 2 | 0 | 1 | 2 | 3 | 4 |  |
| 3 | In the past month, have you felt or behaved as if you went back to the time when the stressful event happened? | 0 | 1 | 2 | 0 | 1 | 2 | 3 | 4 |  |
| 4 | In the past month, have you ever felt uncomfortable when something reminded you of that stressful event? | 0 | 1 | 2 | 0 | 1 | 2 | 3 | 4 |  |
| 5 | In the past month, when you remember the stressful event have you felt any physical change, like, for example, a racing heartbeat, headache, stomachache, difficulty breathing, shivering, or sweating? | 0 | 1 | 2 | 0 | 1 | 2 | 3 | 4 |  |
| 6 | In the past month, have you tried to avoid memories, thoughts, or feelings related to the stressful event you have mentioned (for example, tried not to talk about the event, or tried to forget it or not think about it)? | 0 | 1 | 2 | 0 | 1 | 2 | 3 | 4 |  |
| 7 | In the past month, have you ever tried to distance yourself from the things, people, places, and activities that remind you of the stressful event? | 0 | 1 | 2 | 0 | 1 | 2 | 3 | 4 |  |
| 8 | In the past month, have you ever had difficulty in remembering some essential parts of the stressful event you have mentioned? | 0 | 1 | 2 | 0 | 1 | 2 | 3 | 4 |  |
| 9 | In the past month, have you ever had negative thoughts about yourself, others, life, or the world in general (for example, you thought that you are not a good person, or that people cannot be trusted)? | 0 | 1 | 2 | 0 | 1 | 2 | 3 | 4 |  |
| 10 | In the past month, have you felt the stressful event you have mentioned happened because of your own carelessness or some mistake you made? Or in the last month, have you blamed yourself for part or all of the stressful event you have mentioned? | 0 | 1 | 2 | 0 | 1 | 2 | 3 | 4 |  |
| 11 | In the past month, have you experienced fear or panic, or felt guilt and shame because of the stressful event? | 0 | 1 | 2 | 0 | 1 | 2 | 3 | 4 |  |
| 12 | In the past month, have you felt as though you are no longer able to enjoy the things you used to like in the same way you did before? | 0 | 1 | 2 | 0 | 1 | 2 | 3 | 4 |  |
| 13 | In the past month, have you felt lonely and isolated from people to the extent that you would feel lonely even if you were around other people? | 0 | 1 | 2 | 0 | 1 | 2 | 3 | 4 |  |
| 14 | In the past month, have you been unable to enjoy or express your positive feelings (for example, you felt nothing makes you happy, or you were not able to express love and affection to others such as parents, siblings, friends, and close ones)? | 0 | 1 | 2 | 0 | 1 | 2 | 3 | 4 |  |
| 15 | In the past month, have you gotten angry, argued, or fought because of trivial things? | 0 | 1 | 2 | 0 | 1 | 2 | 3 | 4 |  |
| 16 | In the past month, have you purposely hurt yourself or did something that caused you harm? | 0 | 1 | 2 | 0 | 1 | 2 | 3 | 4 |  |
| 17 | In the past month, you have been vigilant or extremely cautious (for example, you repeatedly look around to know what is going on and who is there)? | 0 | 1 | 2 | 0 | 1 | 2 | 3 | 4 |  |
| 18 | In the past month, have you easily and quickly felt nervous or startled (for example, when you hear a loud sound)? | 0 | 1 | 2 | 0 | 1 | 2 | 3 | 4 |  |
| 19 | In the past month, did you have difficulty concentrating (for example, you are distracted in the classroom, and you were not aware of the teacher or the lesson, or when you were told a story, your mind drifted off)? | 0 | 1 | 2 | 0 | 1 | 2 | 3 | 4 |  |
| 20 | In the past month, have you had trouble sleeping (for example, difficulty sleeping, or you repeatedly woke up)? | 0 | 1 | 2 | 0 | 1 | 2 | 3 | 4 |  |
